# Ultrasensitive Norovirus Detection Using DNA Aptasensor Technology

**DOI:** 10.1371/journal.pone.0079087

**Published:** 2013-11-14

**Authors:** Amanda Giamberardino, Mahmoud Labib, Eman M. Hassan, Jason A. Tetro, Susan Springthorpe, Syed A. Sattar, Maxim V. Berezovski, Maria C. DeRosa

**Affiliations:** 1 Department of Chemistry, Carleton University, Steacie Building, Ottawa, Ontario, Canada; 2 Department of Chemistry, University of Ottawa, Ottawa, Ontario, Canada; 3 Centre for Research on Environmental Microbiology, University of Ottawa, Ottawa, Ontario, Canada; National Institute of Allergy and Infectious Diseases, United States of America

## Abstract

DNA aptamers were developed against murine norovirus (MNV) using SELEX (Systematic Evolution of Ligands by EXponential enrichment). Nine rounds of SELEX led to the discovery of AG3, a promising aptamer with very high affinity for MNV as well as for lab-synthesized capsids of a common human norovirus (HuNoV) outbreak strain (GII.3). Using fluorescence anisotropy, AG3 was found to bind with MNV with affinity in the low picomolar range. The aptamer could cross-react with HuNoV though it was selected against MNV. As compared to a non-specific DNA control sequence, the norovirus-binding affinity of AG3 was about a million-fold higher. In further tests, the aptamer also showed nearly a million-fold higher affinity for the noroviruses than for the feline calicivirus (FCV), a virus similar in size and structure to noroviruses. AG3 was incorporated into a simple electrochemical sensor using a gold nanoparticle-modified screen-printed carbon electrode (GNPs-SPCE). The aptasensor could detect MNV with a limit of detection of approximately 180 virus particles, for possible on-site applications. The lead aptamer candidate and the aptasensor platform show promise for the rapid detection and identification of noroviruses in environmental and clinical samples.

## Introduction

Human noroviruses (HuNoV) are the leading cause of viral gastroenteritis worldwide [1]. Infection can occur sporadically but is more commonly associated with outbreaks [2]. Over 50% of all outbreaks occur in public settings including restaurants, cruise ships and vacation resorts [3]. The virus is endemic in many areas of the world [2], [3], highlighting a need for rapid and accurate testing to ensure the safety of food and water supplies. As HuNoV remain recalcitrant to laboratory culture, they can only be detected through enzyme immunoassays (EIA) or genome amplification [2]. Both methods are complex and expensive, thus of limited field use. Therefore, technologies are needed for rapid, sensitive and accurate detection of HuNoV for possible on-site application.

**Figure 1:**
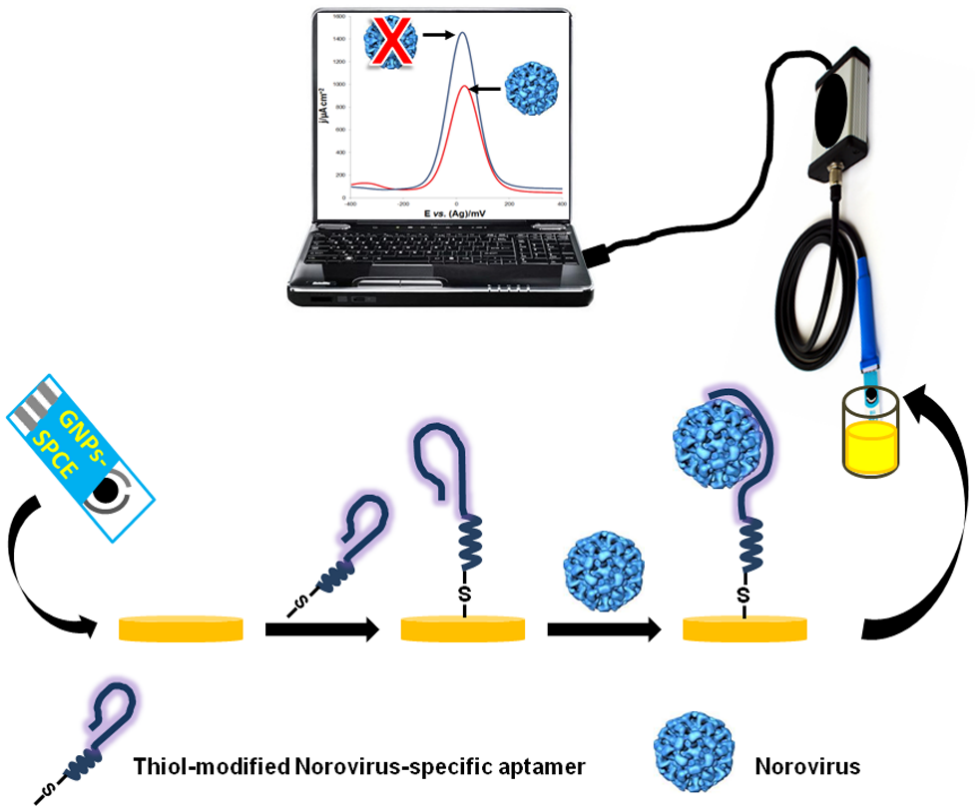
Schematic diagram of the electrochemical detection protocol adopted in this study. A thiolated norovirus-specific DNA aptamer was self-assembled onto a gold nanoparticles-modified screen-printed carbon electrode (GNPs-SPCE). Binding of the virus to the immobilized aptamer causes a decrease in the redox current, measured *via* square wave voltammetry.

Aptamers are synthetic nucleic acids that fold into unique three-dimensional conformations capable of binding a target with remarkable affinity and specificity [4]. In the past three decades, the use of aptamers has grown from detection of small molecules [5] to complex biological entities including cancer cells [6], human pathogenic bacteria [7] and viruses [8]–[9]. We report here the development of aptamers specific to HuNoV and murine norovirus (MNV) using systematic evolution of ligands by exponential enrichment (SELEX) [10], a screening technique that uses iterative rounds of binding, selection and amplification to find binding sequences from highly diverse nucleic acid libraries. A promising aptamer (AG3) was incorporated into an electrochemical aptasensor to detect MNV in solution (Figure 1). The aptasensor is simple and portable enough for field-use without specialized training. This combination of aptamers and biosensing makes it possible to design a kit for the detection of HuNoV in clinical isolates and field samples of food and water for greater public and environmental safety.

## Materials and Methods

### Preparation of Virus Stocks and Reagents

A seed culture of the murine norovirus type 1 (MNV-1; clone CW-1) was received from Dr. H. Virgin, Washington University School of Medicine, St. Louis, MO.

All other virus stocks and culture media were donated by Health Canada and used as received. Samples were dialyzed into selection buffer, herein called general sensing buffer (GSB) 50 mM NaCl, 20 mM Tris-HCl pH 7.4, 3 mM MgCl_2_, 5 mM KCl. Clipped snakeskin tubing and Slide-A-Lyzer cassettes (ThermoFisher Scientific) were prepared according to the manufacturer’s instructions. Briefly, tubing and cassettes containing the virus were incubated in 200 times volume of exchange buffer with stirring, for a minimum of 1 h at room temperature. Buffer was changed and the suspension was allowed to incubate with continuous stirring overnight at room temperature. The equilibrated samples were then concentrated using Amicon Ultracell 50K (50,000 MWCO) filter tubes according to the manufacturer’s instructions. Samples were characterized via A_280_ by either UV-Vis absorption using a Cary Bio300 spectrophotometer with a Starna 50 uL quartz cell, or a ThermoScientific NanoDrop 1000. Extinction coefficients: MNV = 72,560 M^−1^ cm; GII.3 = 47,058 M^−1^ cm; FCV = 105,000 M^−1^ cm. An SDS PAGE gel of the MNV sample used for selections can be found in the (Figure S1 in File S1).

All amidites including 5′Fluorescein phosphoramidite (6-FAM) and hexaethylene glycol spacer (HEGL) for DNA synthesis were purchased from Glen Research. Standard chemical reagents were purchased from Sigma unless otherwise stated. Deionized water obtained through a Milli-Q water purification system (Millipore) was used for all experiments. PCR reagents (Taq Polymerase, 25 mM MgCl_2_, 10 mM dNTP) and PAGE reagents, were purchased from BioShop. All buffers were prepared and filter-sterilized with Corning 0.45 µm cellulose acetate filters before use.

### DNA Synthesis

All sequences were synthesized on a MerMade 6 automated DNA synthesizer via phosphoramidite chemistry, using standard 500 Å controlled pore glass (CPG) columns. Nucleotide sequences were defined as follows: *Library sequence*: 5′-CGT ACG GAA TTC GCT AGC-N_40_-GGA TCC GAG CTC CAC GTG-3′; *Primer 1*∶5′-(6-FAM)-CGT ACG GAA TTC GCT AGC-3′; *Primer 2*∶5′-A_20_-HEGL-CAC GTG GAG CTC GGA TCC-3′; Unmodified primers (herein referred to as Pr1u and Pr2u) have identical nucleotide sequence to Primer 1 and Primer 2 respectively, except without fluorescein or polyA-HEGL modifications. A non-specific DNA sequence, (the thrombin aptamer sequence with additional flanking bases added to create a sequence of similar length and G content) was also prepared for use in controls: 5′-(6-FAM)-TGC TCC TAC AAA TGC CAT CAT TGG TTG GTG TGG TTG GGC TGC AGC GAG CTT ACG-3′. Characterization of sequences was done by UV-VIS and fluoresence spectroscopy. Molecular weight of the AG3 sequence was verified by ESI-LC-MS (Novatia).

### PCR Amplification Procedure

Master mix recipe per PCR reaction consisted of 50 µL of PCR buffer (100 mM KCl, 200 mM Tris, 2% Triton X-100, pH 9), 8 µL of 25 mM MgCl_2_, 2 µL of 10 mM dNTP, 0.5 µL each of 0.2 mM Primer 1 and Primer 2, 1 µL of 500U Taq polymerase and up to 40 µL of deionized water. 5–10 µL of pool template was added to 100 µL aliquots of master mix. Amplification conditions were as follows: 94°C at 5 min (denaturation and heat activation of Taq polymerase); 20 cycles of a) 94°C 1 min, b) 47°C 1 min and c) 72°C 1 min (annealing and extension); 72°C 10 min (final extension); cool at 4°C (end of amplification). Products were resolved by 12% denaturing PAGE.

### SELEX on Murine Norovirus (MNV)

Prior to each round of SELEX, approximately 1–2 nmol of DNA pool in 500 µL of GSB was denatured by heating at 95°C for 5 minutes and allowed to cool for 20 minutes at room temperature. A negative selection was first carried out to reduce non-specific interactions of the pool with the partitioning medium, a 0.45 µm nitrocellulose filter (Millipore HAWP filters). The DNA was re-quantified to determine how much was lost. In the first three rounds of SELEX, the recovered pool was incubated with 0.5 µL of 10^6^ pfu/mL (approximately 500 infectious particles) of MNV for 30 minutes at room temperature and filtered by nitrocellulose to remove non-interacting sequences. The filter was washed 1–2 times with GSB to facilitate removal of non-binding sequences, which were screened in the filtrate and washes via A_260_ measurements. The binding complex was eluted by suspending the filter in 2×500 µL aliquots of elution buffer (7M urea, 50 mM HEPES-NaOH, pH 7.5, 10 mM EDTA) and heating at 95°C for 5 minutes. The binding sequences were purified using phenol-chloroform extraction followed by ethanol precipitation. The pool was desalted with Amicon Y-30K ultrafiltration tubes, as per the manufacturer’s instructions. Binding pool was checked by absorption and fluorescence whenever applicable. The DNA was then amplified by conventional PCR using the abovementioned cycling conditions and visualized by 12% denaturing PAGE. Bands were excised and then eluted by heat shock at 50°C for 5 minutes, 90°C for 5 minutes and incubated with shaking overnight at 37°C. The DNA was purified via ethanol precipitation and desalted for quantification via A_260_ and fluorescence. Rounds 4–7 were carried out as abovementioned with decreasing concentrations of MNV (i.e. approximately 100 infectious particles in Rounds 4 and 5; 10 infectious particles in Rounds 6 and 7). Rounds 8 and 9 were counter-selections with approximately 500 infectious particles of *Feline Calicivirus* (FCV) and an equivalent volume of Dulbecco’s Modified Eagle Medium (DMEM), respectively. The enriched pool was amplified with unmodified primers (Pr1u and Pr2u) for expression in *E. coli.* Cloning was done using the StrataClone® cloning kit, amplified using the TempliPhi® amplification kit (GE Healthcare) as per the manufacturer’s instructions and were sent to University of Calgary’s Core DNA Services for sequencing analysis. Secondary structures were predicted using MFold (http://mfold.rna.albany.edu/).

### Affinity Determination of AG3

Binding characterization of the aptamer candidate (AG3) was conducted via fluorescence anisotropy, using a Horiba Jobin-Yvon Fluorolog spectrophotometer. Briefly, 5′-Fluorescein-modified AG3 sequence was prepared to 1 nM in GSB and denatured as earlier described. 1 in 10 serial dilutions of virus/serum stocks (MNV, GII.3, FCV, DMEM, Grace’s serum) were prepared in GSB. 30 µL of each dilution was mixed with 30 µL of 1 nM AG3 in a microcentrifuge tube and allowed to incubate for ∼1 h at room temperature. All samples were prepared in duplicate. Fluorescence anisotropy was determined on the spectrofluorometer under the following conditions: Excitation: 490 nm, Emission: 520 nm, slit widths: 4.00 nm, Number of trials per sample: 6, Integration time: 1 s. Data was fitted in MS Excel, using a Hill function.

### Polycarbonate Filter Binding Assay

Plates for this filter binding assay were prepared in-house. 0.1 um pore size black polycarbonate filters (AMD Manufacturing Inc.) were adhered onto a black 96-well filter plate (Pall Life Sciences) and left to cure for 24 hrs before use. Samples were prepared by mixing 30 uL of heat-denatured 5′-Fluorescein-modified AG3 (10 nM) with 30 uL of serial dilutions (10^−7^ M-10^−16^ M) of MNV in GSB and incubated for 24 h. Samples in meat juice were prepared in a similar fashion: Meat juice (5 mL) was collected from a package of ground beef (450 g) and mixed thoroughly with one equivalent volume of GSB and filtered via syringe filter (Pall 0.45 um PES), and spiked with MNV. Samples were filtered (∼10 in. Hg) through the polycarbonate filter plate and then analyzed for fluorescence signal using a FluoroMax microplate reader (Horiba Jobin-Yvon). Instrument parameters: lambda excitation = 490 nm, lambda emission = 520 nm, 10 nm excitation and emission slit widths, integration time = 1 s. Fluorescence from filtration of AG3 in GSB or meat juice alone was used as the blank. Data was fitted in Sigma Plot using a Logistic function.

### Preparation of the Aptasensor

Prior to experiments, the gold nanoparticles-modified screen-printed carbon electrode (GNPs-SPCE) (L33×W10×H0.5, Dropsens, Spain) was washed thoroughly with deionized water then dried with N_2_. Subsequently, the electrode was incubated with 500 nM of the HPLC purified HS-5′-AG3 modified at the 5′ position with a 6-hydroxyhexyl disulfide group, detection probe (Integrated DNA Technologies, USA) in 20 mM Tris-ClO_4_ buffer, pH 8.6, for 5 days at 4°C. Finally, the electrode was incubated with 1 mM 2-mercaptoethanol in ethanol for 5 min to back-fill the empty spots of the electrode surface, thus reducing the non-specific adsorption onto the surface.

### Electrochemical Measurements

Electrochemical studies, including cyclic voltammetry (CV), square wave voltammetry (SWV), and electrochemical impedance spectroscopy (EIS) were performed with an electrochemical analyzer (CH Instruments 660D, TX, USA) connected to a personal computer. All measurements were carried out at room temperature in an enclosed and grounded Faraday cage. A conventional three-electrode configuration printed on a ceramic substrate; including a GNPs-SPCE electrode as the working electrode, carbon counter electrode, and a silver pseudo-reference electrode. A three-electric contacts edge connector was used to connect the screen-printed electrode with the potentiostat (Dropsens, Spain). The open-circuit or rest-potential of the system was measured prior to all electrochemical experiments to prevent sudden potential-related changes in the self-assembled monolayer. CV experiments were performed at a scan rate of 100 mV s^−1^ in the potential range from −400 to 800 mV. EIS measurements were conducted in the frequency range of 100 kHz to 0.1 Hz, at a formal potential of 100 mV and AC amplitude of 5 mV. The measured EIS spectra were analyzed with the help of equivalent circuit using ZSimpWin 3.22 (Princeton Applied Research, U.S.) and the data were presented in Nyquist plots. Square wave voltammograms were carried out in the range of −400 to 800 mV with a step potential of 4 mV, amplitude of 5 mV and frequency of 10 Hz. Electrochemical measurements were performed in 25 mM phosphate buffer (pH 7), containing 4 mM K_3_[Fe(CN)_6_] and 10 µM [Ru(NH_3_)_6_]Cl_3_. Importantly, all measurements were repeated for a minimum of three times with separate electrodes. The electro-active area of the GNP-SPCE working electrode was estimated using Randles-Sevčik for quasi-reversible electron transfer processes, I_p_ = 2.69 × 10^5^ *n* ^3/2^ *v* ^1/2^ *D* ^1/2^ *A C,* where *n* is the number of electrons participating in the redox process, *v* is the scan rate, *A* is the area of the electrode, *C* is the concentration of the probe molecule in mol cm,^−3^ and *D* is the diffusion coefficient for 1.0 mM potassium ferrocyanide in 1.0 M KCl (6.3 × 10^−6^ cm^2^ s^−1^). Subsequently, an electroactive area of 0.01 cm^2^ was calculated.

## Results and Discussion

### Aptamer Selection

**Figure 2:**
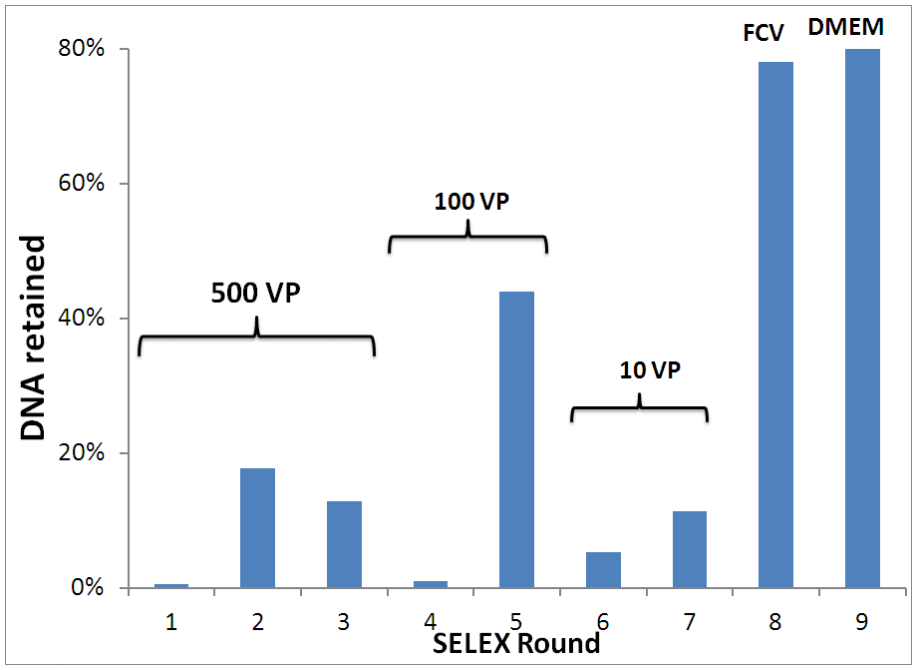
Summary of rounds for MNV SELEX. Target stringencies are outlined for sets of rounds (VP = viral particles). Retained fractions were assessed by fluorescence. DNA retained after counter-selection rounds (8 and 9) represents pool that did not interact with the indicated targets (FCV = feline calicivirus and DMEM = dialyzed components of Dulbecco’s Modified Eagle Medium).

MNV was chosen as the representative target for SELEX since it is often used as a model for HuNoV. MNV can also be readily grown in cell cultures and its infectivity measured. A library of DNA sequences containing a 40-base random region flanked by two defined primer binding regions (5′-CGT ACG GAA TTC GCT AGC-N_40_-GGA TCC GAG CTC CACGTG-3′) was screened for MNV binders using the SELEX method. A negative selection step was first conducted against a nitrocellulose filter, which was used as the partitioning medium in the SELEX experiment. This removed sequences from the starting DNA library that would have a non-specific interaction with the filter. About 1.6 nmol (∼ 1×10^15^ sequences) were passed through the filter with a recovery of 68%. Note that buffer conditions were optimized in order to minimize non-specific binding of the pool to the nitrocellulose membrane. Positive selections then followed using MNV, which was previously dialyzed in general sensing buffer (GSB) before being subjected to SELEX. Selections were run in the presence of approximately 500 infectious particles of MNV, creating a highly competitive environment for the DNA library such that only sequences with a very strong affinity would survive the selection. DNA sequences with affinity for the virus were retained on the nitrocellulose filter, eluted, and PCR-amplified for inclusion in the next selection round. The stringency of the selections was further increased in successive rounds by decreasing target content to ∼100 and 10 infective units, in rounds 4 and 6, respectively, in order to achieve a strongly binding pool. Figure 2 summarizes the observations of the SELEX experiment conducted on MNV. In the first three rounds, a net increase in the amount of retained sequences was observed, supporting the enrichment of the DNA pool from one round to the next. As the number of available targets was reduced to 100 and 10 infective units in subsequent rounds, the fraction of retained sequences initially dropped and then increased dramatically, as expected. In the last two rounds, counter-selections were conducted to eliminate any binders with affinity for related targets. Feline calicivirus (FCV) was chosen for counter-selections as it is from the same family (Caliciviridae) as noroviruses but belongs to the genus Calicivirus with a slightly different capsid structure [11]. A counter-selection was completed with 500 infectious units of FCV and the recovery of DNA that did not interact with the counter-target is shown in Figure 2. A second counter-selection was followed with Dulbecco’s Modified Eagle Medium (DMEM), which is used for culturing MNV in the laboratory, and once again a large recovery of DNA was noted.

After round 9, conventional cloning and sequencing found three potential aptamer candidates out of 40 clones (Table 1). One sequence, herein referred to as AG3, was found with the highest frequency. The sequence was observed to have a mutated forward primer, a slightly truncated random region of 33 nt, as well as a truncated primer region at the 3′ end. Two other clones (AG28 and AG29) maintained their 40 nt random region and intact primer regions. These were comparably different from each other with only 12 conserved nucleotides in the random region. Secondary structure predictions using Mfold [12] gave low complexity structures with stem-loop features common to all three candidates as the lowest energy conformation (Figure S2 in File S1). The unstructured regions could represent binding pockets that interact with the capsid; however, the actual protein-nucleic acid interaction sites remain to be investigated. It is important to note that these structures did not account for G-quadruplex formation, which was considered as a possible secondary structure given observations of the sequences’ high G-content, particularly for AG3 (see Table 1). A QuadFinder [13] analysis showed potential for G-quadruplexes mostly in regions shared by the random region and the primer region (data not shown). Indeed, a DNA melt experiment at 295 nm shows an initial decrease in absorbance with increasing temperature, characteristic of the melting of a quadruplex structure (Figure S3 in File S1) [14]. The T_m_ for this transition, however, is relatively low, approximately 37°C. An increase in absorbance was noted at higher temperature, with a T_m_ of approximately 67°C for this transition. This suggests that both quadruplex and duplex features may make up the structure of the sequence, although which feature is responsible for target binding remains to be seen.

**Table 1:** Summary of aptamer candidates.

Name	Sequence	G content
AG3	GCTAGCGAATTCCGTACGAAGGGCGAATTCCACATTGGGCTGCAGCCCGGGGGATCC	33%
AG28	CGTACGGAATTCGCTAGCACGGGGCTTAAGGAATACAGATGTACTACCGAGCTCATGAGGATCCGAGCTCCACGTG	29%
AG29	CGTACGGAATTCGCTAGCCGACGGTCAATGCTCGTGAGCCAGTACACACAATATATGTGGATCCGAGCTCCACGTG	26%

### Affinity Determination by Fluorescence Anisotropy

**Figure 3:**
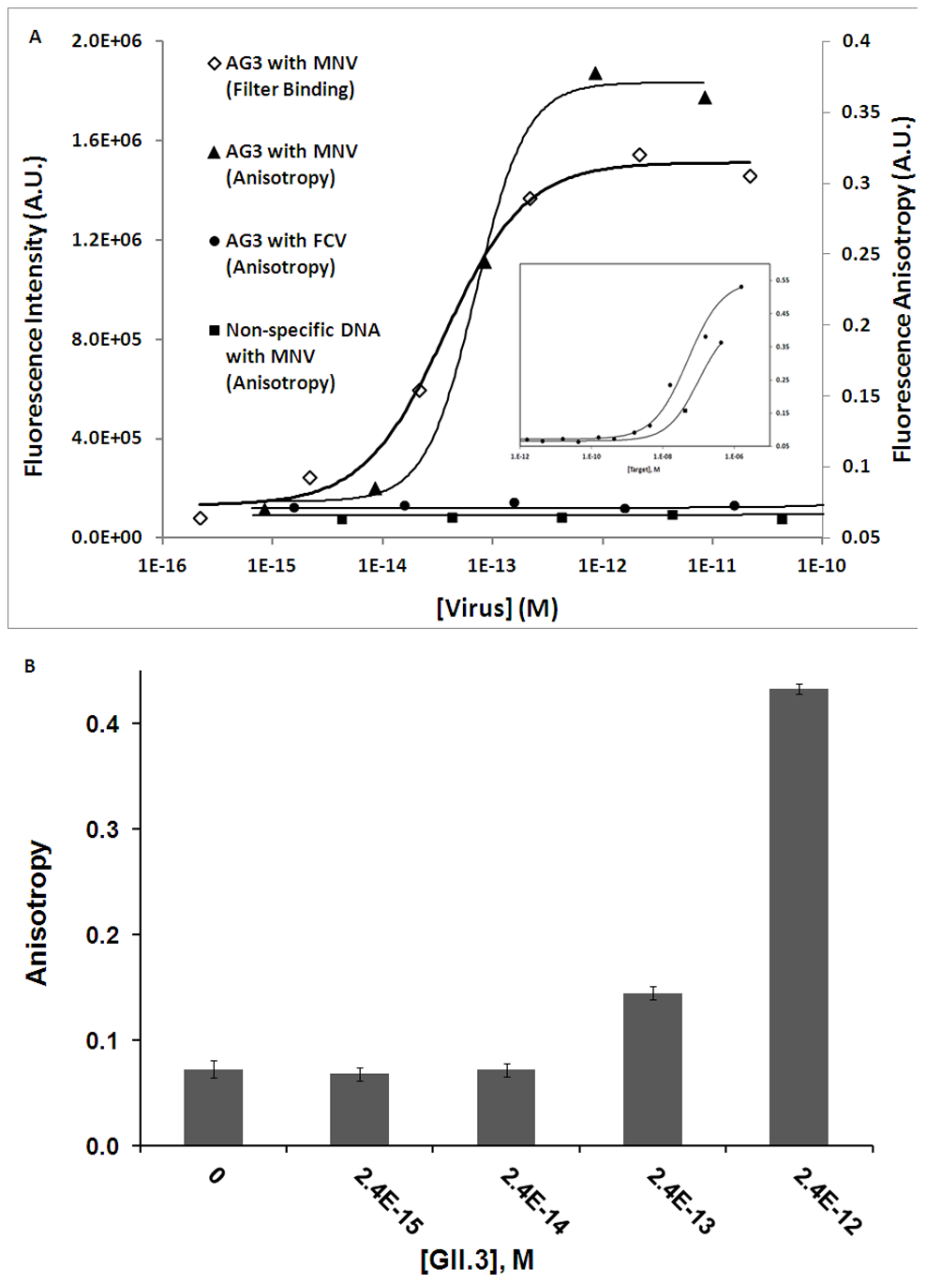
Binding assays. A) Binding affinity measurements for 5′-fluorescein-modified AG3 with MNV using a polycarbonate filter binding assay (diamonds) and a fluorescence anisotropy assay (triangles). The binding affinity of 5′-fluorescein tagged AG3 to FCV (circles) as well as a non-specific DNA control with MNV (squares) were also measured by anisotropy. Measurements are fitted using either the Hill or Logistic functions (solid lines). Inset: Much higher concentrations of virus are required in order to show any anisotropy change in the controls. B) Fluorescence anisotropy results from binding of 5′-fluorescein-modified AG3 with varying concentrations of GII.3 HuNoV capsid.

AG3 was investigated due to its high occurrence among sequenced clones. Fluorescence anisotropy was chosen to assess target binding. Figure 3A shows the binding curves for fluorescein-tagged AG3 with various targets at a range of concentrations. Among the targets studied, AG3 showed excellent affinity for MNV with an apparent dissociation constant (EC50) in the low picomolar range. A search of the Aptamerbase [15] puts AG3 in the high affinity range for aptamers discovered to date, particularly in comparison to aptamers for other pathogenic targets. For example, the aptamer selected against *Campylobacter jejuni* has a K_d_ of approximately 290 nM [16]. Note that the viral capsid is composed of 180 VP1 capsid proteins, thus there is a high likelihood that this is not a monovalent K_d_ and that an effective “avidity” is contributing to the apparent binding strength of the aptamer. In this case, this effect is not caused by the use of multimeric aptamers but rather the presence of multiple copies of the target in close proximity, thus allowing a dissociated aptamer to rapidly re-bind to a nearby capsid protein.

AG3 was also found to be selective for noroviruses over other targets. Anisotropy was used to estimate the binding affinity of AG3 for FCV, and the apparent dissociation constant is in the low micromolar range (see Figure 3A and inset). Furthermore, DMEM and Grace’s Insect Media showed only minimal effects on the anisotropy of the tagged AG3 (Figure S4 in File S1) suggesting limited non-specific binding. It is important to note that in the cases of these counter targets, saturation of the anisotropy signal was not observed at the concentrations tested. Thus, the estimated dissociation constants may even be overestimating the weak binding affinity of the aptamer for these non-specific targets. In order to confirm that virus binding of AG3 is sequence dependent, the binding affinity of MNV to a non-specific oligonucleotide sequence (the sequence is that of the thrombin aptamer flanked with extra nucleotides to yield a sequence of similar length and composition) was also tested (Figure 3A and inset). The non-specific sequence showed drastically reduced binding affinity compared to the AG3 sequence (again estimated in the low micromolar range). Thus, the binding affinity of AG3 is not limited to a non-specific interaction between the DNA and the capsid proteins.

To test whether this aptamer candidate could be applied to the detection of HuNoV, fluorescence anisotropy was used to assess the binding of AG3 to the lab-synthesized capsid of a common HuNoV outbreak strain, GII.3. AG3 showed high affinity for the HuNoV capsid (Figure 3B), with a clear change in anisotropy detected at concentrations as low as 240 fM. Due to the limited availability of the GII.3 capsid sample, a high enough concentration was not available in order to confirm signal saturation, limiting our ability to determine an accurate EC50 or apparent K_d_. Nevertheless, this aptamer candidate displays considerable binding affinity for one strain of HuNoV.

### Polycarbonate Filter Binding Assays

To further confirm the binding affinity of our aptamer, we attempted to perform a nitrocellulose filter binding assay using 5′-fluorescein-labelled AG3. This proved difficult as the background fluorescence of the nitrocellulose membrane reduced the sensitivity of our measurements (data not shown). Thus, black polycarbonate filter plates were prepared in-house and were used as the membrane for our binding assay to eliminate autofluorescence. Varying concentrations of MNV in either GSB or 50% meat juice in GSB were incubated with 5′-fluorescein-modified AG3 for 24 h and filtered through the 96-well plate. In GSB, (Figure 3A) the filter assay results paralleled what was observed in the anisotropy, with an apparent EC50 in the subpicomolar range (ca. 0.1 pM). Fluorescence quenching was noted at concentrations of MNV higher than 0.1 nM. Preliminary filter binding experiments for MNV spiked in 1∶1 meat juice/GSB solutions were also attempted (see Figure S5). Meat juice extracted from retail ground beef was filter sterilized, diluted by half with GSB, and spiked with low concentrations of MNV. The solutions were incubated with 5′-fluorescein-labelled AG3 and then filtered through the polycarbonate filter plate. The wells with the filtered meat juice solution alone showed low background fluorescence comparable to the polycarbonate filter alone. The spiked samples were compared to AG3 alone and virus; sub-picomolar concentrations could be detected in this complex matrix. Fluorescence quenching at higher MNV concentrations were noted in the meat juice samples as well; further investigation into this is ongoing. Nevertheless, AG3 proved promising enough to proceed to aptasensor development.

### Development of the Electrochemical Aptasensor

Based on the high affinity of AG3 for noroviruses, a simple and inexpensive electrochemical aptasensor was developed. A 5′-thiol-modified AG3 self-assembled monolayer on a gold nanoparticles-modified screen-printed carbon electrode served as the sensor platform. The attractive features of this approach were the ease of preparation and potential portability of this sensor.

**Figure 4:**
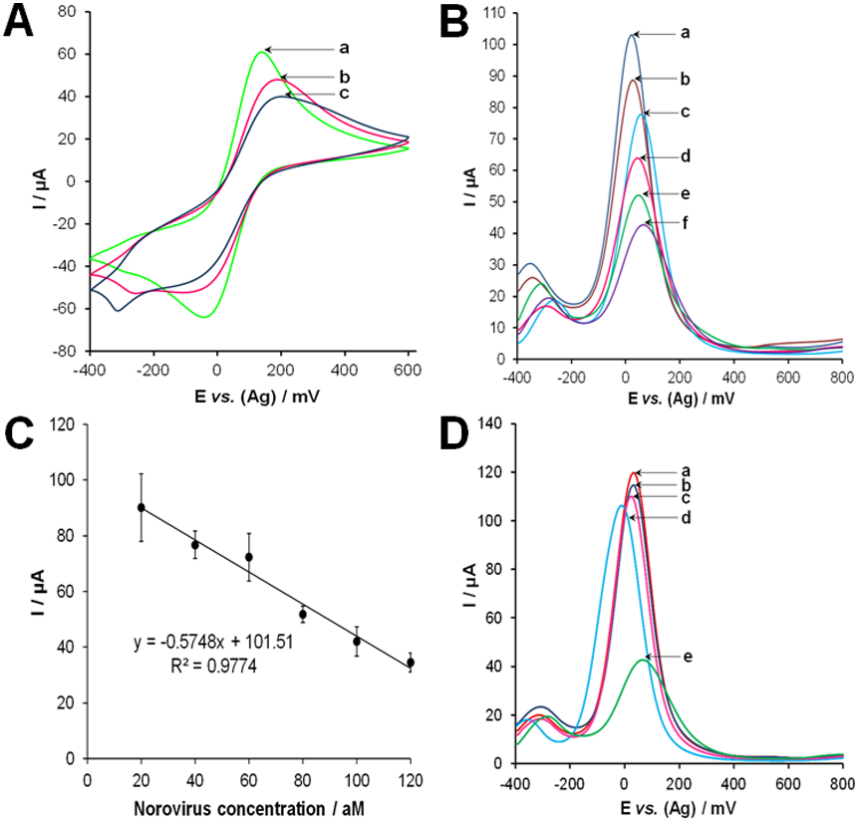
Aptasensor results. (A) Cyclic voltammograms of the norovirus aptasensor after each immobilization or binding step. The [Fe(CN)_6_]^3−/2−^ redox couple was monitored for these experiments and cyclic voltammograms were recorded at a scan rate of 100 mV s^,–1^ where (*a*) bare SPGE; (*b*) after self-assembly of the thiolated norovirus specific aptamer; (*c)* after back-filling with 1 mM 2-mercaptoethanol. (B) Square wave voltammograms obtained using (*a*) 20 aM, (*b*) 40 aM, (*c*) 60 aM, (*d*) 80 aM, (*e*) 100 aM, and (*f*) 120 aM of norovirus in buffer. (C) Calibration plot of current *vs.* concentration of norovirus. (D) Selectivity experiments performed using (a) buffer alones, (b) 5000 PFU of vesicular stomatitis virus, (c) 5.1 mg mL^−1^ HSA, (d) 5000 PFU of vaccinia virus (e) 120 aM of norovirus. All experiments were performed in Dulbecco’s phosphate buffered saline after incubation with the developed aptasensor for 1 hr at 25°C. Square wave voltammograms were carried out in the range of −400 to 800 mV with a step potential of 4 mV, amplitude of 5 mV and frequency of 10 Hz. Electrochemical measurements were performed in 25 mM phosphate buffer (pH 7), containing 4 mM K_3_[Fe(CN)_6_] and 10 µM hexaamine ruthenium chloride.

The electrochemical characteristics of the aptasensor during assembly were investigated using cyclic voltammetry (CV) examining the [Fe(CN)_6_]^3−/2−^ redox couple. The untreated gold electrode presents a quasi-reversible voltammogram indicating that the redox active probe had easy access to the bare gold surface, as evidenced by very large redox currents (*curve a*) shown in Fig. 4A. Formation of a self-assembled monolayer of the thiolated aptamer onto the gold surface substantially reduced the electrode current (curve b), indicating the formation of a more compact blocking layer. The concentration of the aptamer (500 nM) was optimized to maximize surface density. Final treatment with 2-mercaptoethanol further reduced the redox current because it can penetrate down to the electrode surface, thereby blocking direct access of the conducting ions (*curve c*). The thiol group of the back-filling agent can effectively displace the weaker adsorption contacts between the aptamer nucleotides and the gold surface, leaving the capture probe tethered primarily through the thiol end group. Such conformation improves the flexibility of the probe and renders it more accessible for target binding. Moreover, displacement of the non-specific adsorption provides free volume into the film, which enhances the transport of counter ions and solvent molecules through the modified film [17].

The ferricyanide/ruthenium hexaammine reporter system, previously described elsewhere [18], [19], was applied to facilitate high-sensitivity readout of virus detection. The approach relies on the primary electron acceptor [Ru(NH_3_)_6_]^3+^, which is attracted electrostatically to the electrode surface at levels that are correlated with the amount of bound aptamers. The inclusion of [Fe(CN)_6_]^3−^ during the electrochemical measurement serves to regenerate the Ru^III^ substrate, as the Fe^III^ species is easier to reduce. Subsequently, the Fe^III^ is repelled electrostatically from the electrode and hence primarily undergoes chemical reduction by Ru^II^. Admittedly, some direct electrochemical reduction of Fe^III^ is expected to occur. Indeed, the signals generated by our sensor correlate well with what has been previously described for this reporter system in a low density DNA film-based sensor [20].

### Analytical Parameters of the Developed Aptasensor

Aliquots of varying concentrations of MNV (20, 40, 60, 80, 100, 120 aM) in 30 µL of Dulbecco’s phosphate buffered saline were incubated with the aptasensor at 25°C for 1 h. Square wave voltammetry (SWV) was performed at each concentration and it was observed that that binding between the virus and the immobilized aptamer causes a decrease in the current intensity. Hence, the modulation of the electrochemical signal was recorded as a function of the current intensity (*I*). This could be interpreted as a further blocking of the surface upon binding of the bulky virus to the surface-bound aptamers. As shown in Fig. 4B and C, the *I* value decreases linearly with increasing concentration of norovirus, in the range from 20 aM to 120 aM (ca. 360 to 2170 viral particles), with the regression equation of y = −0.5748x +101.51 (R^2^ = 0.9774), where *y* is the *I* value in µA and *x* is the concentration of norovirus in aM. The relative standard deviation (RSD) values were between 5.6% and 13.5%. Beyond the upper norovirus level, the response became nonlinear, indicating the saturation of the surface with the virus. The limit of detection (LOD) was 10 aM (ca. 180 viral particles), estimated from 3(*S_b_*/*m*), where *S_b_* is the standard deviation of the measurement signal for the blank and *m* is the slope of the analytical curve in the linear region. A parallel analysis was performed using electrochemical impedance spectroscopy (EIS), as described in Results S1 and shown in Figure S6, which confirmed the electrochemical findings from SWV. It was observed that the charge transfer resistance (*R_CT_*) value, which can be used to monitor the binding event [21], increases linearly with increasing concentration of norovirus, in the range from 20 aM to 120 aM. The regression equation was y = 19.147x +1129.2 (R^2^ = 0.9928), where *y* is the *R_CT_* value in Ω and *x* is the concentration of norovirus in aM, as shown in Fig. S5B and Table S1. As shown in Fig. 4D, the selectivity of the developed aptasensor was tested using 5000 plaque forming units (PFU) of vesicular stomatitis virus (VSV) in place of norovirus and it resulted in only a 9.1% decrease in current intensity (103.7 µA) when compared with buffer treatment (0%, 110.6 µA) and 120 aM (2170 viral particles) of norovirus (100%, 34.6 µA). Furthermore, using 5000 PFU of vaccinia virus caused only an 11.9% decrease in *I* value (101.5 µA). Finally, the specificity of the sensor was also tested with 5.1 mg mL^−1^ (∼77 µM) human serum albumin (HSA, Sigma-Aldrich, USA) which caused a 15% decrease in *I* value (99.2 µA). Thus, both the high affinity and the selectivity of the aptamer is reflected in the aptasensor’s response to noroviruses and to non-specific targets.

### Implications

The focus of this preliminary investigation was HuNoV, which can spread by various environmental means to cause sporadic cases as well as frequent outbreaks in domestic and institutional settings. HuNoVs also remain refractory to culture in the laboratory, thus precluding their recovery from clinical and environmental samples as infectious virions. While immunological and molecular means may be applied in clinical samples, generally low levels of viral contamination in samples of food and water, along with the presence of interfering substances, make detection of such viruses much more challenging. The estimated infectious dose of norovirus ranges from 18–1000 virus particles [22]. The median fecal load of noroviruses depends on the genotype but it could be as low as 10^5^ particles per gram of stool [23]. Thus, the combination of our high-affinity aptamer sequence and electrochemical sensing technology may be sensitive enough to be applied directly to clinical samples and also possibly to environmental samples, either directly or after a modest concentration step. This is the direction of our current efforts. The use of aptamers offers a potential alternative in the development of sensitive, specific and relatively inexpensive field kits to rapidly detect the presence of HuNoV.

## Conclusions

This is the first report of the development and application of aptamers for detection of noroviruses. After 9 rounds, aptamer AG3 was selected and found to recognize both MNV and the HuNoV capsid GII.3. AG3 was also proven to be selective for the noroviruses over other viruses (FCV as shown by fluorescence anisotropy, VSV and vaccinia as demonstrated by electrochemistry). Based on the results of this study, the aptasensor with the aptamer AG3 has demonstrated proof-of-concept for use in norovirus detection. Future studies with a variety of matrices are still required to ensure that the aptasensor will be effective in the numerous environments suspected of contamination with noroviruses. Moreover, the use of the aptasensor in the field will need to be tested to ensure that a biosensor will be not only simple but also practical. In addition, this study opens other potential applications of AG3 through functionalization of magnetic beads for capture methods, enzymatic probes for visual identification and surface immobilization for lab-on-a-chip purposes.

## Supporting Information

File S1 SDS PAGE gel of the MNV target used in selections, Mfold predicted secondary structures, UV melting and fluorescence data, filter binding data, as well as impedance studies. This material is available free of charge via the Internet at http://pubs.acs.org.
(DOCX)
