# Supplementary material for: Ultrasensitive Norovirus Detection Using DNA Aptasensor Technology
Source: PLoS One. 2013 Nov 14;8(11):e79087. doi: 10.1371/journal.pone.0079087 (PMC3828344; doi:10.1371/journal.pone.0079087)
Supplement: File S1 — SDS PAGE gel of the MNV target used in selections, Mfold predicted secondary structures, UV melting and fluorescence data, filter binding data, as well as impedance studies. This material is available free of charge via the Internet at http://pubs.acs.org. (DOCX) [file pone.0079087.s001.docx]

Supporting information for:

Ultrasensitive norovirus detection using DNA aptasensor technology.

Amanda Giamberardino, Mahmoud Labib, Eman M. Hassan, Jason Tetro, Susan Springthorpe, Syed Sattar, Maxim V. Berezovski and Maria C. DeRosa.

**
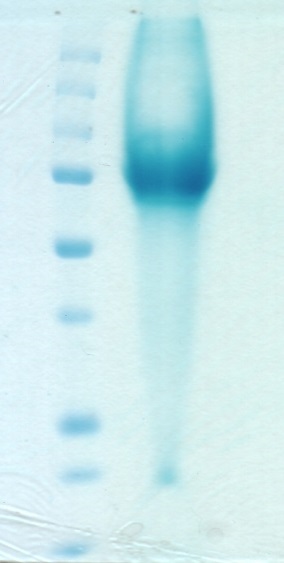
**

**Figure S1: SDS PAGE gel of the MNV preparation used for selection.**

**AG3**

**AG28**

**AG29**

**Figure S2: MFold secondary structure predictions of aptamer candidate sequences.**

**Figure S3: UV melting temperature (T_m_) experiment. The absorbance at 295 nm is monitored as the temperature is increased from 20 to 80°C. Loss of absorbance is typically associated with G-quartet melting while an increase in absorbance is suggestive of the melting of duplex structure.**

**
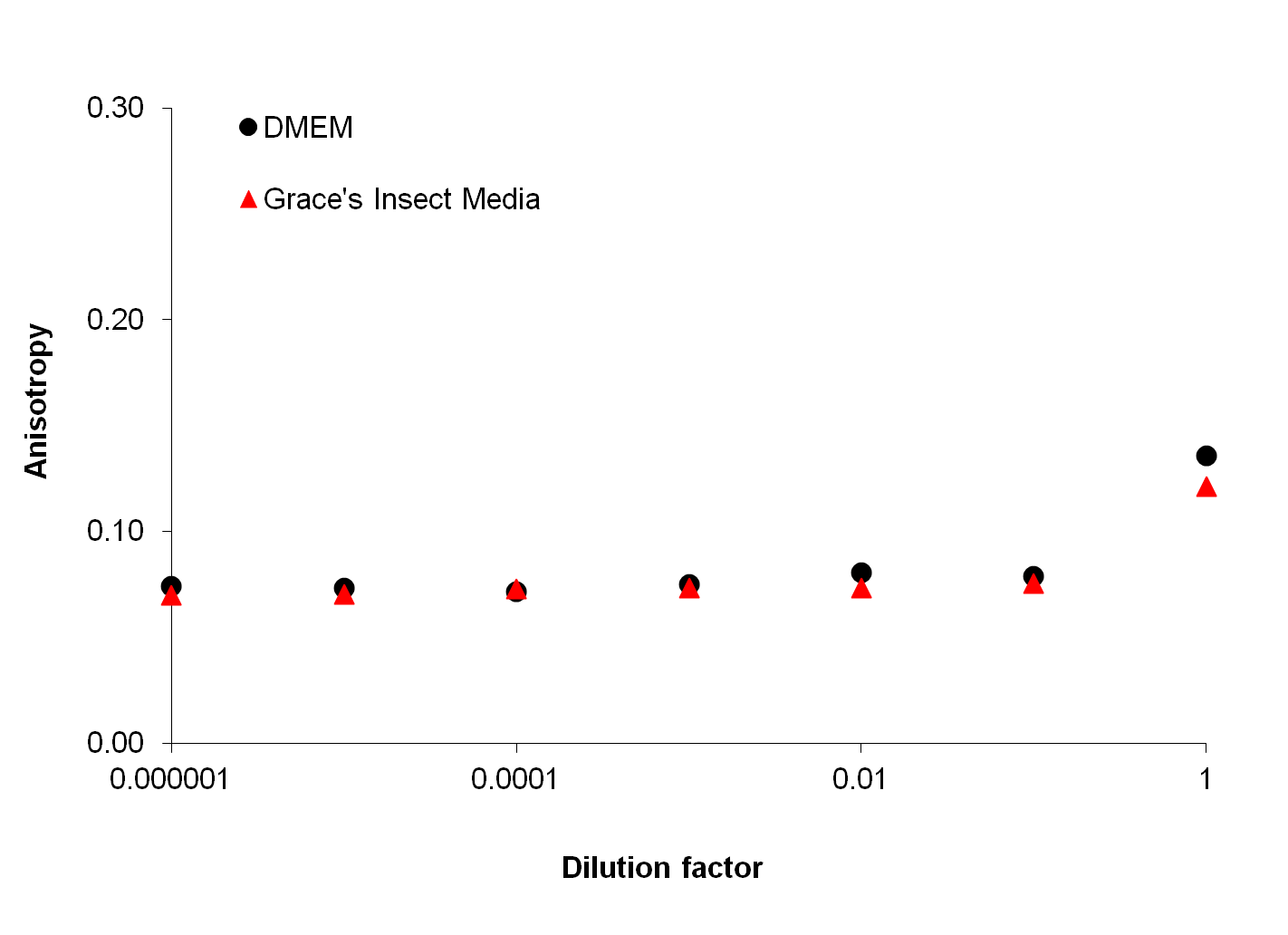
**

**Figure S4: Fluoresence anisotropy plots of media controls DMEM (circles) and Grace’s Insect Media (triangles). Even concentrated media showed little effect on the anisotropy of 5’-fluorescein labeled AG3.**

**Figure S5: Fluorescence data from filter binding experiment (n=2) with AG3 and MNV in meat juice. 5’-Fluorescein labelled AG3 was mixed with 1:1 meat juice/GSB either alone (AG3) or spiked with MNV (1.08E-14 M; 1.08E-15M; 1.08E-16M) and filtered on a black polycarbonate 96 well filter plate prepared in-house. The fluorescent signal was compared to that from filtering meat juice alone (meat juice).**

**Results S1: Impedance studies**

The complex impedance was presented as the sum of the real Z, *Z_re_*, and imaginary Z, *Z_im_*, components that originate mainly from the resistance and capacitance of the cell, respectively. A suitable equivalent circuit, shown in the inset of **Fig. S6A**, was carefully selected to reflect the real electrochemical process and to enable a fit producing accurate values. A modified Randles circuit consists of the ohmic resistance; *R_S_*, of the electrolyte solution, the electronic charge transfer resistance, *R_CT_*, in series with the finite length Warburg *W*, and in parallel with a constant phase element, *CPE*, associated with the double layer and reflects the interface between the assembled film and the electrolyte solution. The solution resistance, *R_S_*, is the resistance between the aptamer-modified electrode and the reference electrode. The high frequency semicircle of the Nyquist diagram corresponds to the charge transfer resistance, *R_CT_*, in parallel with the *CPE*. The former represents the electron-transfer kinetics of the redox probe at the electrode surface, whereas the latter corresponds to a nonlinear capacitor accounting for the inhomogeneity of the formed film [1]. The diameter of the semicircle corresponds to the interfacial resistance at the electrode surface, the value of which depends on the dielectric and insulating features of the surface layer. On the other hand, the Warburg impedance, *Z_W_*, accounts for a diffusion-limited electrochemical process, presumably due to molecular motions within the film caused by the penetration of conducting ions [2]. The rationale behind this electrochemical approach is that the binding between the target norovirus and its respective aptamer will further block the charge transfer from a solution-based redox probe to the electrode surface. Consequently, *R_CT_* will become increasingly large and can be used to monitor the binding event.

(1) Dijksma, M.; Boukamp, B. A.; Kamp, B.; van Bennekom, W. P. *Langmuir* **2002**, *18*, 3105-3112.

(2) Baur, J.; Gondran, C.; Holzinger, M.; Defrancq, E.; Perrot, H.; Cosnier, S. *Anal Chem* **2010**, *82*, 1066-1072.


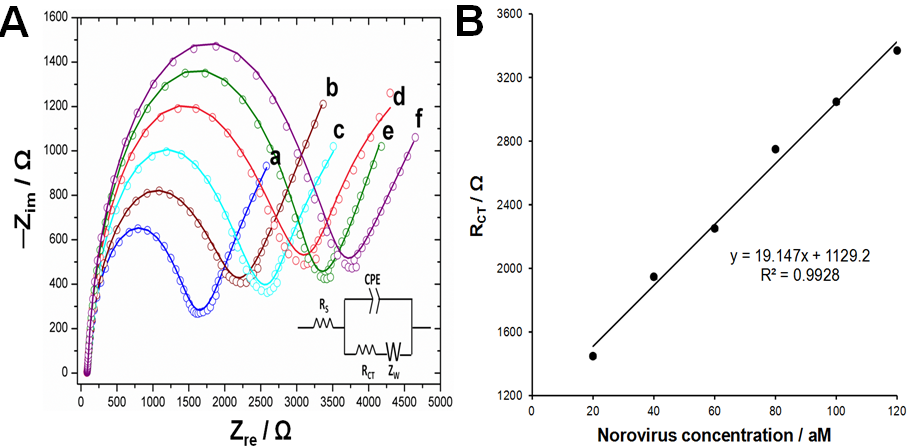


**Figure S6.** **(A)** Nyquist plot (*─Z_im_* *vs.* *Z_re_*) of impedance spectra obtained using (*a*) 20 aM, (*b*) 40 aM, (*c*) 60 aM, (*d*) 80 aM, (*e*) 100 aM, and (*f*) 120 aM of norovirus in buffer. The impedance spectra were recorded from 100 kHz to 0.1 Hz and the amplitude was 0.1 V *vs.* Ag. **(B)** Calibration plot of resistance to charge transfer (*R_CT_*) *vs.* concentration of norovirus. Electrochemical measurements were performed in 25mM phosphate buffer (pH 7), containing 4 mM K_3_[Fe(CN)_6_] and 10 µM [Ru(NH_3_)_6_]Cl_3_.

**Table S1.** Equivalent circuit element values for the developed norovirus aptasensor, in the presence of increasing concentrations of norovirus

| Concentration of the virus (aM) | *Rs (Ω)* | *CPE* (µF) | *n* | *R_CT_ (Ω)* | *W* (µF^0.5^) |
| --- | --- | --- | --- | --- | --- |
| 20 | 87.44 | 2.46 | 0.91 | 1449 | 892 |
| 40 | 87.13 | 3.07 | 0.88 | 1948 | 688.9 |
| 60 | 89.02 | 2.38 | 0.91 | 2252 | 725.7 |
| 80 | 86.15 | 2.66 | 0.91 | 2750 | 665.2 |
| 100 | 89.38 | 2.35 | 0.92 | 3048 | 876 |
| 120 | 88.21 | 2.17 | 0.92 | 3370 | 791.9 |
